# Next generation sequencing in cancer research and clinical application

**DOI:** 10.1186/1480-9222-15-4

**Published:** 2013-02-13

**Authors:** Derek Shyr, Qi Liu

**Affiliations:** 1 Washington University, 63130, St. Louis, MO, USA; 2 Center for Quantitative Sciences, Vanderbilt University School of Medicine, 37232, Nashville, TN, USA; 3 Department of Biomedical Informatics, Vanderbilt University School of Medicine, 37232, Nashville, TN, USA

**Keywords:** Next generation sequencing, Cancer research, Clinical application

## Abstract

The wide application of next-generation sequencing (NGS), mainly through whole genome, exome and transcriptome sequencing, provides a high-resolution and global view of the cancer genome. Coupled with powerful bioinformatics tools, NGS promises to revolutionize cancer research, diagnosis and therapy. In this paper, we review the recent advances in NGS-based cancer genomic research as well as clinical application, summarize the current integrative oncogenomic projects, resources and computational algorithms, and discuss the challenge and future directions in the research and clinical application of cancer genomic sequencing.

## Introduction

Sanger sequencing has dominated the genomic research for the past two decades and achieved a number of significant accomplishments including the completion of human genome sequence, which made the identification of single gene disorders and the detection of targeted somatic mutation for clinical molecular diagnostics possible [1,2]. Despite Sanger sequencing's accomplishments, researchers are demanding for faster and more economical sequencing, which has led to the emergence of “next-generation” sequencing technologies (NGS). NGS’s ability to produce an enormous volume of data at a low price [3,4] has allowed researchers to characterize the molecular landscape of diverse cancer types and has led to dramatic advances in cancer genomic studies.

**Figure 1:**
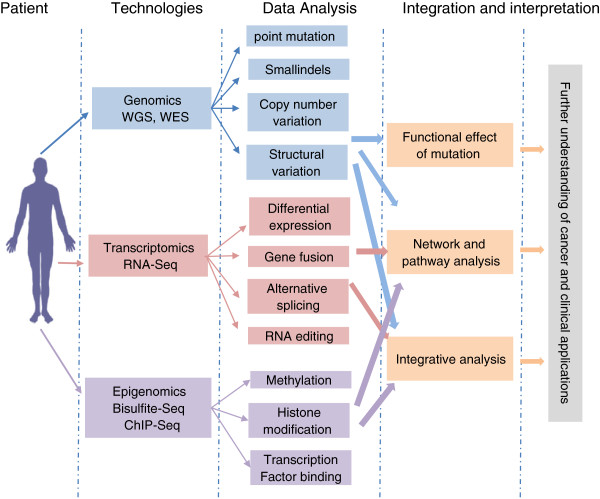
**The workflow of integrating omics data in cancer research and clinical application.** NGS technologies detect the genomic, transcriptomic and epigenomic alternations including mutations, copy number variations, structural variants, differentially expressed genes, fusion transcripts, DNA methylation change, etc. Various kinds of bioinformatics tools are used to analyze, integrate, and interpret the data to improve our understanding of cancer biology and develop personalized treatment strategy.

The application of NGS, mainly through whole-genome (WGS) and whole-exome technologies (WES), has produced an explosion in the context and complexity of cancer genomic alterations, including point mutations, small insertions or deletions, copy number alternations and structural variations. By comparing these alterations to matched normal samples, researchers have been able to distinguish two categories of variants: somatic and germ line. The Whole transcriptome approach (RNA-Seq) can not only quantify gene expression profiles, but also detect alternative splicing, RNA editing and fusion transcripts. In addition, epigenetic alterations, DNA methylation change and histone modifications can be studied using other sequencing approaches including Bisulfite-Seq and ChIP-seq. The combination of these NGS technologies provides a high-resolution and global view of the cancer genome. Using powerful bioinformatics tools, researchers aim to decipher the huge amount of data to improve our understanding of cancer biology and to develop personalized treatment strategy. Figure 1 shows the workflow of integrating omics data in cancer research and clinical application.

## Cancer research

In the last several years, many NGS-based studies have been carried out to provide a comprehensive molecular characterization of cancers, to identify novel genetic alterations contributing to oncogenesis, cancer progression and metastasis, and to study tumor complexity, heterogeneity and evolution. These efforts have yielded significant achievements for breast cancer [5-12], ovarian cancer [13], colorectal cancer [14,15], lung cancer [16], liver cancer [17], kidney cancer [18], head and neck cancer [19], melanoma [20], acute myeloid leukemia (AML) [21,22], etc. Table 1 summarizes the recent advances in cancer genomics research applying NGS technologies.

**Table 1:** Recent NGS-based studies in cancer

**Cancer**	**Experiment Design**	**Description**	**ref**
Colon cancer	72 WES, 68 RNA-seq, 2 WGS	Identify multiple gene fusions such as RSPO2 and RSPO3 from RNA-seq that may function in tumorigenesis	[15]
Breast cancer	65 WGS/WES, 80 RNA-seq	36% of the mutations found in the study were expressed. Identify the abundance of clonal frequencies in an epithelial tumor subtype	[11]
Hepatocellular carcinoma	1 WGS, 1 WES	Identify TSC1 nonsense substitution in subpopulation of tumor cells, intra-tumor heterogeneity, several chromosomal rearrangements, and patterns in somatic substitutions	[17]
Breast cancer	510 WES	Identify two novel protein-expression-defined subgroups and novel subtype-associated mutations	[5]
Colon and rectal cancer	224 WES, 97 WGS	24 genes were found to be significantly mutated in both cancers. Similar patterns in genomic alterations were found in colon and rectum cancers	[14]
squamous cell lung cancer	178 WES, 19 WGS, 178 RNA-seq, 158 miRNA-seq	Identify significantly altered pathways including NFE2L2 and KEAP1 and potential therapeutic targets	[16]
Ovarian carcinoma	316 WES	Discover that most high-grade serous ovarian cancer contain TP53 mutations and recurrent somatic mutations in 9 genes	[13]
Melanoma	25 WGS	Identify a significantly mutated gene, PREX2 and obtain a comprehensive genomic view of melanoma	[20]
Acute myeloid leukemia	8 WGS	Identify mutations in relapsed genome and compare it to primary tumor. Discover two major clonal evolution patterns	[21]
Breast cancer	24 WGS	Highlights the diversity of somatic rearrangements and analyzes rearrangement patterns related to DNA maintenance	[8]
Breast cancer	31 WES, 46 WGS	Identify eighteen significant mutated genes and correlate clinical features of oestrogen-receptor-positive breast cancer with somatic alterations	[7]
Breast cancer	103 WES, 17 WGS	Identify recurrent mutation in CBFB transcription factor gene and deletion of RUNX1. Also found recurrent MAGI3-AKT3 fusion in triple-negative breast cancer	[6]
Breast cancer	100 WES	Identify somatic copy number changes and mutations in the coding exons. Found new driver mutations in a few cancer genes	[9]
Acute myeloid leukemia	24 WGS	Discover that most mutations in AML genomes are caused by random events in hematopoietic stem/progenitor cells and not by an initiating mutation	[22]
Breast cancer	21 WGS	Depict the life history of breast cancer using algorithms and sequencing technologies to analyze subclonal diversification	[12]
Head and neck squamous cell carcinoma	32 WES	Identify mutation in NOTCH1 that may function as an oncogene	[19]
Renal carcinoma	30 WES	Examine intra-tumor heterogeneity reveal branch evolutionary tumor growth	[18]

### Discovery of new cancer-related genes

Cancer is primarily caused by the accumulation of genetic alterations, which may be inherited in the germ line or acquired somatically during a cell’s life cycle. The effects of these alterations in oncogenes, tumor suppressor genes or DNA repair genes, allows cells to escape growth and regulatory control mechanisms, leading to the development of a tumor [23]. The progeny of the cancer cell may also undergo further mutations, resulting in clonal expansion [24]. As clonal expansion continues, clones eventually become invasive to its surrounding tissue and metastasize to distant areas from the primary tumor [25].

The sequencing of cancer genomes has revealed a number of novel cancer-related genes, especially in breast cancer. Recently, six papers reported their findings on large breast cancer dataset: TCGA performed exome sequencing on 510 samples from 507 patients [5], Banerji et al. conducted exome sequencing on 103 samples and whole genome sequencing on 17 samples, Ellis et al. did exome sequencing on 31 samples and whole genome sequencing on 46 samples [7], Stephens et al. applied exome sequencing on 100 samples, Shah et al. performed whole genome/exome and RNA sequencing on 65 and 80 samples of triple-negative breast cancers [11], and Nik-Zainal et al. performed whole genome sequencing on 21 tumor/normal pairs [12]. Besides confirming recurrent somatic mutations in TP53, GATA3 and PIK3CA, these studies discovered novel cancer-related mutations. Although novel mutations occur at low frequency (less than 10%), mutations of specific genes are enriched in the subtype of breast cancers and could be grouped into cancer-related pathways. For example, mutations of MAP3K1 frequently occur in luminal A subtype [5,7]. Pathways involving p53, chromatin remodeling and ERBB signaling are overrepresented in mutated genes [11]. Furthermore, some mutations indicate therapeutic opportunities such as the mutant GATA3, which might be a positive predictive marker for aromatase inhibitor response [7].

Genomic sequencing has also helped characterize the mutation profile of colorectal cancer. For example, exome sequencing performed on 72 tumor-normal pairs identified 36,303 protein-altering somatic mutations. Further analysis for significantly mutated genes led to 23 candidates that included expected cancer genes such as KRAS, TP53 and PIK3CA and novel genes such as ATM, which regulates the cell cycle checkpoint. RNA sequencing identified recurrent R-spondin fusions, which might potentiate Wnt signaling and induce tumorigenesis [15]. Another example includes exome sequencing performed on 224 tumor and normal pairs. This study identified 15 highly mutated genes in the hypermutated cancers and 17 in the non-hypermutated cancers. Among the non-hypermutated cancers, novel frequent mutations in SOX9, ARID1A, ATM and FAM123B were detected besides the known APC, TP53 and KRAS mutations. The analysis of the mutations and functional roles of SOX9, ARID1A, ATM and FAM123B suggested they are highly potential colorectal cancer-related genes. Non-hypermutated colon and rectum cancers were found to have similar patterns in genomic alternation. Whole genome sequencing of 97 tumors with matched normal samples identified the recurrent NAV2-TCF7L1 fusion [14].

### Tumor heterogeneity and evolution

What makes cancer a difficult disease to conquer has much to do with the evolution of cancer that results from the selection and genetic instability occurring in each clone, leading to heterogeneity in tumors [26]. This idea was first proposed by Peter Nowell in 1976 as the clonal evolution model of cancer, which attempted to explain the increase in tumor aggressiveness over a period of time. Further work by other researchers in the 1980s supported this theory with studies of metastatic subclones from a mouse sarcoma cell line [26].

The wide application of NGS has revealed substantial insights into tumor heterogeneity and tumor evolution. Variations between tumors are referred to as intertumor heterogeneity, while variations within a single tumor are intratumor heterogeneity. Intertumor heterogeneity is recognized by different morphological phenotype, expression profiles and mutation and copy number variation patterns, categorizing tumors into different subtypes [27-31]. The mRNA-expression subtype was found to be associated with somatic mutation landscapes in the recent TCGA and Eillis et al.’s studies. [5,7]. As a huge amount of somatic mutations generated by NGS, the picture emerges like that individual tumor is unique, each containing distinct mutation patterns. For instance, Stephens et al. found that there were 73 different combination possibilities of mutated cancer genes among the 100 breast cancers [9].

Intratumor heterogeneity can be recognized as non-identical cellular clones or subclones within a single tumor, indicating different histology, gene expression, and metastatic and proliferative potential. The ability to generate high-resolution data makes NGS a particularly useful tool for studying intratumor heterogeneity. A recent NGS-based study on renal cell carcinoma from four patients has successfully illuminated intratumor heterogeneity [18]. For patient 1, the pre-treatment samples of the primary tumor and chest-wall metastasis went through exon-capture multi-region sequencing on DNA. Of the 128 validated mutations found in 9 regions of the primary tumor, 40 were ubiquitous, 59 were shared by some regions, and 29 were unique to specific regions, showing that genetic heterogeneity exists within a tumor and an “ongoing regional clonal evolution” [18]. Most importantly, the study showed that a single biopsy of a tumor only reveals a small part of a tumor’s mutational landscape; from a single biopsy, about 55% of all mutations were detected in this tumor and 34% were shared by most regions of the tumor.

The ongoing and parallel evolution of cancer cells may establish and maintain intratumor heterogeneity. For example, phylogenetic relationships of the tumor regions in patient 1 and 2 by the renal cell carcinoma study revealed a branching rather than linear evolution of the tumor [18]. Studies have also shown branching structures of evolution in breast cancer [26]. According to the “Trunk-Branch Model of Tumor Growth” [26], there are somatic events that promote tumor growth, which represents the trunk of the tree in the early stage of tumor development. These somatic aberrations would most likely be ubiquitous at this stage. Over time, other somatic events, known as drivers, cause tumor heterogeneity to occur, which causes branching to take place in tumors as well as in metastatic sites. Later, these branches will evolve and become more isolated, resulting in a ‘Bottleneck Effect’ that can result in chromosomal instability, allowing further expansion of tumor heterogeneity [26]. This leads to the tumor’s ability to adapt and survive in changing environments, which affects the success of drug treatment [18]. Therefore, it is important to examine tumor clonal structure and identify common mutations located in the trunk of the phylogenetic tree, which may help understand target therapy resistance and discover more robust therapeutic approaches.

## Clinical application

Besides allowing researchers to understand mutations in cancer, NGS has already been applied to the clinic in many areas including prenatal diagnostics, pathogen detection, genetic mutations, and more [32]. Although genetic mutations have been identified with Sanger sequencing, PCR, and microarrays in clinical application, these three have limitations that don’t apply to NGS. For example, although microarrays can detect single nucleotide variants (SNVs), they have trouble identifying larger DNA aberrations, e.g., large indels and structural rearrangements, which are common in cancer. In contrast, whole exome and whole-genome sequencing can provide the clinician a comprehensive view of the DNA aberrations, genetic recombination, and other mutations [28,32]. Therefore, NGS platforms serve as a good diagnostic and prognostic tool and help clinicians identify specific characteristics in each patient, paving the road towards personalized medicine.

NGS has already been applied in the clinic for cancer diagnosis and prognosis. For example, whole genome sequencing identified a novel insertional fusion that created a classic bcr3 PML-RARA fusion gene for a patient with acute myeloid leukemia and the findings altered the treatment plan for the patient [33]. By sequencing the tumor genome of a patient, clinicians are able to design patient-specific probes that uses DNA in the patient’s blood serum to monitor the progress of a patient’s treatment and detect for any signs of relapse [27-31]. The discovery of more biomarkers and the development of target-therapies will be essential in helping a clinician choose the best personalized treatment for his or her patients.

There has also been a dramatic increase in the number of clinical trials using NGS technologies since 2010 (Table 2). Ranging from WGS and WES to RNA-seq and targeted sequencing, clinical trials are using NGS to find genetic alterations that are the drivers of certain diseases in patients and apply that knowledge into the practice of clinical medicine. The information gained from these studies may help with drug development and explain the resistance of certain treatments.

**Table 2:** Active cancer studies using NGS as the primary outcome measure

**Study Title/*Sponsor***	**NCT#/# Enrolled/Start Date**	**Condition**	**Description**	**Sequencing Technologies**
Tumor Specific Plasma DNA in Breast Cancer/*Dartmouth-Hitchcock Medical Center*	NCT01617915/6/October 2012	Breast Cancer	Analyze chromosomal rearrangements and genomic alterations	Whole genome sequencing
Whole Exon Sequencing of Down Syndrome Acute Myeloid Leukemia*/Children’s Oncology Group*	NCT01507441/10/February 2012	Leukemia	Examine DNA samples of patients with Leukemia and Down Syndrome and identify DNA alterations	Whole exome Sequencing
Studying Genes in Samples From Younger Patients with Adrenocortical Tumor/*Children’s Oncology Group*	NCT01528956/10/February 2012	Adrenocortical Carcinoma	Study genes from patients with adrenocortical tumor	Whole genome Sequencing
Feasibility Clinical Study of Targeted and Genome-Wide Sequencing/*University Health Network, Toronto*	NCT01345513/150/March 2011	Solid Tumors	Identify gene mutations in cancer patients	Whole genome sequencing
An Ancillary Pilot Trial Using Whole Genome Sequencing in Patients with Advance Refractor Cancer/*Scottsdale Healthcare*	NCT01443390/10/September 2011	Advanced Cancer	Investigate patients with cancer that are using Phase I drugs and its effect on the patient	Whole genome Sequencing
Cancer Genome Analysis/*Seoul National University Hospital*	NCT01458604/100/August 2011	Malignant Tumor	Identify and analyze genetic alterations in tumors for therapeutic agents	Targeted Sequencing, whole exome sequencing and RNA-seq
RNA Biomarkers in Tissue Samples From Infants with Acute Meyloid Leukemia/*Children’s Oncology Group*	NCT01229124/20/October 2010	Leukemia	Analyze tissue samples and identify biomarkers from RNA	RNA-seq
Molecular Analysis of Solid Tumors/*St. Jude Children’s Research Hospital*	NCT01050296/360/January 2010	Pediatric Solid Tumors	Analyze gene expression profiles of tumor and examine genetic alterations	Whole genome Sequencing
Deep Sequencing of the Breast Cancer Transcriptome/*University of Arkansas*	NCT01141530/30/Sept 2009	Breast Cancer	Examine transcriptional regulation and triple negative breast cancer	RNA-seq

### Methods and resources

#### Pipeline and tools for NGS data analysis

To analyze and interpret the increasing amount of sequencing data, a number of statistical methods and bioinformatics tools have been developed. For WGS and WES, the analysis generally includes read alignment, variant detection (point mutation, small indels, copy number variation and structural rearrangement) and variant functional prediction (Table 3). Reads are mapped back to the human reference genomes using MAQ [34], BWA [35,36], Bowtie2 [37], BFAST [38], SOAP2 [39], Novoalign/NovoalignCS, SSAHA2 [40], SHRiMP [41], etc. These methods differ in their computational efficiency, sensitivity and ability to accurately map noisy reads, to deal with long or short reads and pair-end reads. Having aligned the reads to the genome, mutation calling identifies the sites in which at least one of the bases differs from a reference sequence by GATK [42], SAMtools [43], SOAPsnp [44], SNVMix [45], Varscan [46], etc. Differing in the underlying statistical models, the performances of these methods are comparable and vary on sequencing depths [47-49]. Detecting somatic mutation involves mutation calling in paired tumor-normal DNA, coupled with comparison to the reference. A naïve somatic mutation caller applies standard calling tools on the normal and tumor samples separately and then selects mutations detected in tumor but not in normal. Alternatively, a complicated caller jointly analyzes tumor-normal pair data such as Varscan2 [50], Somaticsniper [51] and JointSNVMix [52]. SIFT [53], PolyPhen [54], CHASM [55] and ANNOVAR [56] have been developed to understand the impact of the mutations on gene function and to distinguish between driver and passenger mutations. For WGS, various kinds of structural variations can be discovered using BreakDancer [57], VariationHunter [58], PEMer [59] and SVDetect [60]. RNA-seq data analysis generally includes reads alignment, gene expression quantification, differentially expressed genes/isoforms or alternative splicing detection and novel transcripts discovery (Table 4). There are two major approaches to map RNA-seq reads. One is to align reads to the reference transcriptome using standard DNA-seq reads aligner. The alternative is to map reads to the reference genome allowing for the identification of novel splice junctions using a RNA-seq specific aligner, such as TopHat [61], MapSplice [62], SpliceMap [63], GSNAP [64], and STAR [65]. Having aligned reads, expression values are quantified by aggregating reads into counts and differential expression analysis is performed based on counts (DEseq [66],edgeR [67]) or FPKM/RPKM values (CuffLinks [68,69]). Estimating isoform-level expression is very difficult since many genes have multiple isoforms and most reads are shared by different isoforms. To deal with read assignment uncertainty, Alexa-seq [70] counts only the reads that map uniquely to a single isoform, while Cufflinks [68,69] and MISO [71] construct a likelihood model that best explains all the reads obtained in the experiment. In addition, fusion transcripts can be detected using SOAPfusion, TopHat-Fusion [72], BreakFusion [73], FusionHunter [74], deFuse [75], FusionAnalyser [76], etc. To obtain a more complete view of cancer genome, an integrative approach to study diverse mutations, transcriptomes and epigenomes simultaneously on the pathways or networks is much more informative and promising. A growing number of pathway-oriented tools is now becoming available, including PARADIGM [77], NetBox [78], MEMo [79], CONEXIC [80], etc.

**Table 3:** Computational tools for cancer genomics

**Category**	**Program**	**URL**	**Ref**
Alignment	MAQ	http://maq.sourceforge.net/	[34]
	BWA	http://bio-bwa.sourceforge.net/	[35,36]
	Bowtie2	http://bowtie-bio.sourceforge.net/bowtie2/	[37]
	BFAST	http://bfast.sourceforge.net	[38]
	SOAP2	http://soap.genomics.org.cn/soapaligner.html	[39]
	Novoalign/NovoalignCS	http://www.novocraft.com/	
	SSAHA2	http://www.sanger.ac.uk/resources/software/ssaha2/	[40]
	SHRiMP	http://compbio.cs.toronto.edu/shrimp/	[41]
Mutation calling	GATK	http://www.broadinstitute.org/gatk/	[42]
	Samtools	http://samtools.sourceforge.net/	[43]
	SOAPsnp	http://soap.genomics.org.cn/soapsnp.html	[44]
	SNVmix	http://compbio.bccrc.ca/software/snvmix/	[45]
	VarScan	http://varscan.sourceforge.net/	[46,50]
	Somaticsniper	http://gmt.genome.wustl.edu/somatic-sniper/	[51]
	JointSNVMix	http://compbio.bccrc.ca/software/jointsnvmix/	[52]
SV detection	BreakDancer	http://breakdancer.sourceforge.net/	[57]
	VariationHunter	http://variationhunter.sourceforge.net/	[58]
	PEMer	http://sv.gersteinlab.org/pemer/	[59]
	SVDetect	http://svdetect.sourceforge.net/	[60]
Function effect of mutation	SIFT	http://sift.jcvi.org/	[53]
	CHASM	http://wiki.chasmsoftware.org	[55]
	PolyPhen-2	http://genetics.bwh.harvard.edu/pph2/	[54]
	ANNOVAR	http://www.openbioinformatics.org/annovar/	[56]

Source: http://www.clinicaltrials.gov.

**Table 4:** Computational tools for cancer transcriptomics

**Category**	**Program**	**URL**	**ref**
Spliced alignment	TopHat	http://tophat.cbcb.umd.edu/	[61,69]
	MapSplice	http://www.netlab.uky.edu/p/bioinfo/MapSplice	[62]
	SpliceMap	http://www.stanford.edu/group/wonglab/SpliceMap/	[63]
	GSNAP	http://research-pub.gene.com/gmap/	[64]
	STAR	http://gingeraslab.cshl.edu/STAR/	[65]
Differential expression	CuffDiff	http://cufflinks.cbcb.umd.edu/	[68,69]
	EdgeR	http://www.bioconductor.org/packages/2.11/bioc/html/edgeR.html	[67]
	DESeq	http://www-huber.embl.de/users/anders/DESeq/	[66]
	Myrna	http://bowtie-bio.sourceforge.net/myrna/index.shtml	[81]
Alternative splicing	CuffDiff	http://cufflinks.cbcb.umd.edu/	[68,69]
	MISO	http://genes.mit.edu/burgelab/miso/	[71]
	DEXseq	http://watson.nci.nih.gov/bioc_mirror/packages/2.9/bioc/html/DEXSeq.html	[82]
	Alexa-seq	http://www.alexaplatform.org/alexa_seq/	[70]
Gene fusion	SOAPfusion	http://soap.genomics.org.cn/SOAPfusion.html	
	TopHat-Fusion	http://tophat.cbcb.umd.edu/fusion_index.html	[72]
	BreakFusion	http://bioinformatics.mdanderson.org/main/BreakFusion	[73]
	FusionHunter	http://bioen-compbio.bioen.illinois.edu/FusionHunter/	[74]
	deFuse	http://sourceforge.net/apps/mediawiki/defuse/	[75]
	FusionAnalyser	http://www.ilte-cml.org/FusionAnalyser/	[76]

### Comprehensive cancer projects and resources

The vast amount of oncogenomics data are generated from large scale collaborative cancer projects (Table 5). The Cancer Genome Atlas (TCGA) and International Cancer Genome Consortium (ICGC) are the two largest representatives of such coordinated efforts. Beginning as a three-year pilot in 2006, TCGA aims to comprehensively map the important genomic changes that occur in the major types and subtypes of cancer. TCGA will examine over 11,000 samples for 20 cancer types (http://cancergenome.nih.gov/). ICGC launched in 2008 and its goal is ‘to obtain a comprehensive description of genomic, transcriptomic and epigenomic changes in 50 different tumor types and/or subtypes which are of clinical and societal importance across the globe’(http://icgc.org/icgc). The Cancer Genome Project (CGP) has many efforts at the Sanger Institute and aims to identify sequence variants/mutations critical in the development of human cancers (http://www.sanger.ac.uk/genetics/CGP/). The NCI’s Cancer Genome Anatomy Project (CGAP) seeks to determine the gene expression profiles of normal, precancer and cancer cells, leading eventually to improved detection, diagnosis and treatment for the patient (http://cgap.nci.nih.gov/). Recently, the Clinical Proteomic Tumor Analysis Consortium (CPTAC) has launched to systematically identify proteins that derive from alterations in cancer genomes using proteomic technologies (http://proteomics.cancer.gov/). The combination of genomic and proteomic initiatives is anticipated to produce a more comprehensive inventory of the detectable proteins in a tumor and advance our understanding of cancer biology.

**Table 5:** Comprehensive cancer projects and resources

**Name**	**Description**	**URL**
Comprehensive cancer projects		
The Cancer Genome Atlas	A joint effort to accelerate our understanding of the molecular basis of cancer through the application of genome analysis technologies	http://cancergenome.nih.gov/
International Cancer Genome Consortium	International consortium with the goal of obtaining comprehensive description of genomic, transcriptomic, and epigenomic changes in 50 different cancer types and/or subtypes of clinical and societal importance across the globe	http://icgc.org/icgc
Cancer Genome Anatomy Project	Interdisciplinary program to determine the gene expression profiles of normal, precancer, and cancer cells, leading eventually to improved detection, diagnosis, and treatment for the patient	http://cgap.nci.nih.gov/
Cancer Genome Project	To identify somatically acquired sequence variants/mutations and hence identify genes critical in the development of human cancers	http://www.sanger.ac.uk/genetics/CGP/
The Clinical Proteomic Tumor Analysis Consortium	A comprehensive and coordinated effort to accelerate the understanding of the molecular basis of cancer through the application of proteomic technologies	http://proteomics.cancer.gov/
Resources		
COSMIC	Catalogue of Somatic Mutations in Cancer	http://www.sanger.ac.uk/genetics/CGP/cosmic/
Progenetix	Copy number abnormalities in human cancer from CGH experiments	http://www.progenetix.org/cgi-bin/pgHome.cgi
MethyCancer	An information resource and analysis platform for study interplay of DNA methylation, gene expression and cancer	http://methycancer.psych.ac.cn/
IntOGen	Integrates multidimensional OncoGenomics Data for the identification of genes and groups of genes involved in cancer development	http://www.intogen.org/
Oncomine	A cancer microarray database and integrated data-mining platform	http://www.oncomine.org/
cBio	Provides visualization, analysis and download of large-scale cancer genomics data sets	http://www.cbioportal.org/
Firehose	Provides L3 data and L4 analyses packaged in a form amenable to immediate algorithmic analysis	https://confluence.broadinstitute.org/display/GDAC/Home
UCSC Cancer Genomics Browser	A suite of web-based tools to visualize, integrate and analyze cancer genomics and its associated clinical data	https://genome-cancer.soe.ucsc.edu/
Cancer Genome Workbench	Hosts mutation, copy number, expression, and methylation data from a number of projects, including TCGA, TARGET, COSMIC, GSK, NCI60. It has tools for visualizing sample-level genomic and transcription alterations in various cancers.	https://cgwb.nci.nih.gov/

The data and the results from these projects are freely available to the research community (Table 5). A number of databases and frameworks have been developed to make the data and the results easily and directly accessible. For example, the results from CGP are collated and stored in http://COSMIC[83]. The cBio Cancer Genomics Portal, containing dataset from TCGA and published papers, is specifically designed to interactively explore multidimensional cancer genomics data, including mutation, copy number variations, expression changes (microarray and RNA-seq), DNA methylation values, and protein and phosphoprotein levels [84]. Intogen is also a framework that facilitates the analysis and integration of multimensional data for the identification of genes and biological modules critical in cancer development [85]. The Broad GDAC Firehose, designed to coordinate the various tools utilized by TCGA, provides level 3 and level 4 analyses and enables researchers to easily incorporate TCGA data into their projects. Table 5 also includes resources useful for cancer research but not built on NGS data, e.g., Progenetix [86].

## Challenges and perspective

Although NGS has already helped researchers discover a plethora of information in the field of cancer, challenges in translating the large amounts of oncogenomics data into information that can be easily interpretable and accessible for cancer care still lie ahead. From a computational point of view, many technical and statistical issues remain unsolved. For example, repetitive DNA represents a major obstacle for the accuracy of read alignment and assembly, as well as structure variation detection [87]. Furthermore, it is difficult to distinguish rare mutations in tumor from sequencing and alignment artifacts, especially when a tumor has low purity. Despite new methods to comprehensively catalogue genomic variants, the prediction of their functional effect and the identification of disease-causal variants are still in an early phase [88]. Current algorithms for quantifying isoform expression are not computationally trivial and are incredibly difficult to explain. Although the concept of integrative analysis is not new, predictive networks or pathway models that combine various omics data are still underway. Most importantly, since sequencing technologies and methodologies are both evolving rapidly, it is a difficult challenge to store, analyze and present the data in a method that is transparent and reproducible [89]. On the other hand, tumor complexity and heterogeneity make the analysis and the interpretation of sequencing data even harder. Heterogeneity is dynamic and evolves over time. This challenges the simple notion of binning mutations as tumorigenesis ‘driver’ and neutral ‘passenger’, since some passengers are also drivers just waiting for the right context [90].

From a clinical point of view, a major challenge is to assess genomic variants as potential therapeutic targets. Although many diverse variants are demonstrated to converge on similar deregulated pathways, there is still a lack of pathway-targeted therapies. With the discovery of intra-tumor heterogeneity, questions have been raised about how well a glimpse of a tumor’s genomic landscape can steer the treatment. Currently, many clinicians decide a treatment based on the genetic markers from a few biopsies. Whether these markers are over- or under-represented in the tumor is unknown, causing the selection of treatment to be difficult [29]. In addition to heterogeneity, the tumor’s ability to evolve allows it to have more opportunities to adapt and survive to various treatments. Some researchers hope that with current target therapies, intratumor heterogeneity will decrease to a certain point [29] so that clinicians can then target the non-responsive clones before a tumor re-growth and more mutations can occur; however, choosing an appropriate target therapy will be a challenge. A few researchers have already shown certain treatments, such as the cytotoxic therapies, that have increased genome instability and diversity, resulting in a faster tumor evolution rate and, thus, heterogeneity. The fact is that this area of cancer is understudied [26]; however, one of the key challenges researchers must solve is identifying branched subclones are resistant to which target therapies. More knowledge of network medicine and the interaction between the trunk and branch mutations may lead to appropriate target therapies and personalized therapeutic strategies that can prevent drug resistance and effectively eradicate cancer [26,91].

To accelerate the rate of translating genomic data into clinical practice, a sustained collaboration among multiple centers and effective communication among bioinformaticians, statistical geneticists, molecular biologists and physician are required. Bioinformaticians and statistical geneticists are responsible for providing reproducible and accurate analysis, identifying ‘drivers’ in the unstable and evolving cancer genome and building powerful and flexible integrative model to consider interactions among genomic, transcriptomic, metabolomics, proteomics and epigenomic alterations in the context of tumor microenvironment. Biologists interpret and confirm the functional relevance of variants to cancer. Physicians assess relationships of variants to cancer prognosis and response to therapy. Appropriate infrastructure within each research institution that integrates the clinic for patient samples, wet lab for sequencing, and Bioinformatics for data analysis should allow the sequenced data to be processed efficiently, producing results that can create effective personalized therapies applicable to the clinic. In addition, easily accessible and understandable databases that connect genomic findings with clinical outcome are also required. With these efforts and developments, NGS will greatly potentiate genome-based cancer diagnosis and personalized treatment strategies.

## Competing interests

The authors declare that they have no competing interests.

## Authors’ contributions

QL led the project. DS drafted the manuscript and QL revised the manuscript. All authors read and approved the final manuscript.
